# Slight induction and strong inhibition of *Heterodera glycines* hatching by short-chain molecules released by different plant species

**DOI:** 10.21307/jofnem-2021-071

**Published:** 2021-07-30

**Authors:** Jeanny A. Velloso, Vicente P. Campos, Willian C. Terra, Aline F. Barros, Márcio P. Pedroso, Luma A. Pedroso, Letícia L. Paula

**Affiliations:** 1 Laboratory of Nematology, Department of Plant Pathology, Universidade Federal de Lavras – UFLA, Lavras, Minas Gerais, Brazil; 2 Department of Chemistry, Universidade Federal de Lavras – UFLA, Lavras, Minas Gerais, Brazil

**Keywords:** Ecology, *Glycine max*, Hatching, Soybean Cyst Nematode, Volatile organic compounds

## Abstract

New management tools are necessary to reduce the damage caused by the soybean cyst nematode (SCN), *Heterodera glycines*. Identification of molecules that can stimulate second-stage juveniles (J2) hatching in an environment without food may contribute to that. In in vitro experiments, we evaluate the effect of volatile organic compounds (VOCs) released by soybean (*Glycine max*), bean (*Phaseolus vulgaris*), ryegrass (*Lolium multiflorum*), and alfalfa (*Medicago sativa*) on *H. glycines* egg hatching. VOCs released by all plant species significantly (*p* < 0.05) increased egg hatching. Short-chain molecules released by leaves and roots of soybean and bean increased the hatching up to 71.4%. The analysis of the volatilome done by gas chromatography coupled with mass spectrometry revealed 44 compounds in the plant emissions. Four of them, namely 3-octanol, 1-hexanol, hexanal and linalool were tested individually as hatching inductors. Under concentrations of 200, 600, and 1,000 µg/ml there was no hatching induction of *H. glycines* J2 by these compounds. On the other hand, in these concentrations, the compounds 3-octanol and 1-hexanol caused hatching reduction with values similar to the commercial nematicide carbofuran (2,3-dihydro-2,2-dimethylbenzofuran-7-yl methyl carbamate). In subsequent tests, the compounds 1-hexanol and 3-octanol showed lethal concentration values required to kill 50% of thenematode population (LC_50_) of 210 and 228 µg/ml, respectively, in the first experiment and, 230 and 124 µg/mlin the second one. Although we have not identified any molecules acting as hatching factor (HF), here we present a list (44 candidate molecules) that can be explored in future studies to find an efficient HF.

Soybean (*Glycine max* (L.) Merr) is an important source of proteins and oil, and therefore, more research are essential to increase productivity under different conditions, including biotic stress (Pagano and Miransari, 2016). The soybean cyst nematode (SCN, *Heterodera glycines*) is one of the main pathogens causing yield reduction worldwide and, just in the United States it is responsible for losses of more than U$1 billion annually (Koenning and Wrather, 2010; Wu et al., 2019). The planting of resistant cultivars has still been the most effective SCN control strategy. However, the continued use of the same sources of resistance has selected SCN virulent populations (Yan and Baidoo, 2018). Thus, new management strategies that seek to reduce the damage caused by this pathogen are necessary.

*Heterodera glycines* is a sedentary endoparasite whose life cycle begins with cell multiplication and embryogenesis within the egg, followed by four juvenile stages (J1 to J4) up to the adult stage (reproductive).Within this process, the infective stage J2 needs to count on its lipid reserves that are limited, to guarantee its survival until it finds their host plant (Wharton, 2004). Cyst nematodes, such as *H. glycines*, developed a sophisticated parasite-host interaction, in which the egg hatching is dependent on the presence of hatching factors released by their host, thus synchronizing hatching and availability of food (Perry, 2002; Thapa et al., 2017).

Over the past decades, some of SCN hatching factors have been identified, e.g., glycinoeclipines A, B, and C isolated from aqueous root extracts of bean (*Phaseolus vulgaris*) (Fukusawa et al., 1985; Masamune et al., 1987). Zinc chloride (ZnCl_2_) is also an inducer of the *H. glycines* hatching whose action occurs due to the presence of enzymes dependent on Zn^2+^ that are involved in the permeability of eggshells (Perry et al., 2013; Tefft and Bone, 1984). Soybean and non-host plants such as, ryegrass (*Lolium multiflorum* Lam.) and alfalfa (*Medicago sativa* L.) also released chemical molecules that induce *H. glycines* J2 hatching (Riga et al., 2001). However, these chemical compounds have not yet been fully determined. The identification of new SCN hatching factors released by the root system can assist in the formulation of new control methods (Wang et al., 2018). For example, the pre-planting application of hatching inducers would stimulate the J2 hatching in an environment without food, resulting in their death. This strategy, called ‘suicide hatching’ was proposed by Devine and Jones (2000a). Devine and Jones (2001) also reported significant induction of hatching on field population of *Globodera rostochiensis* by the incorporation of tomato root exudates in the soil. Over the last few years, studies have provided new insights into the ‘suicide hatching’ strategy, which in the future may be useful for farmers to control cyst nematodes in the field (Gautier et al., 2020a, b; Ngala et al., 2021).

Nonetheless, the studies that sought to identify SCN hatching factors concentrated on compounds from root extracts or exudates (Fukusawa et al., 1985; Masamune et al., 1987; Riga et al., 2001) without investigating the role of short-chain molecules, such as the volatile organic compounds (VOCs). Although studies on the effects of VOCs on hatching are scarce, the nematicidal activity of these molecules have received more attention (Aissani et al., 2015; Barros et al., 2014; Silva et al., 2018, 2019). In addition, many of them have the potential to be used as soil biofumigants (Gomes et al., 2020; Ntalli et al., 2018; Pedroso et al., 2019).

Soybean, bean, ryegrass, and alfalfa plants have exudates and root extracts that stimulate the hatching of the SCN (Riga et al., 2001). In this paper, we investigated the role of VOCs released by these plants as *H. glycines* hatching factors.

## Materials and methods

### Nematode culture and J2 collection

The isolate of *H. glycines* race 3 was multiplied on soybean plants cv. M6410 IPRO (Monsoy^®^) in ceramic pots in the greenhouse. Cysts were collected from the roots and soil according to the technique of Shepherd (1970) with modifications. For the extraction of the cysts, the roots were carefully separated from the soil. Then, they were placed on an 850-µm sieve coupled to a 150-µm sieve and subjected to a strong jet of tap water. In this way, the cysts were retained in the 150-µm sieve. For the extraction of cysts present in the soil, 200 cm^3^ of soil were placed in a cup where water was then added until completing one liter. After 1 min of agitation on the soil shaker, the suspension was poured into an 850-µm sieve coupled to the 150-µm sieve. The cysts were retained in the 150-µm sieve. To obtain the eggs, a set of sieves with an opening of 150, 75, and 25 µm was used. The cysts were deposited on a 150-µm sieve and pressed mechanically with a flat base small beaker and washed with tap water jets. Eggs retained on a 25-μm sieve were transferred to a hatching chamber made from a Petri dish (9 cm diameter) housing a 25-μm sieve (5 cm diameter). Then the hatching chamber was transferred to an incubator at 27 ± 2°C. Second-stage juveniles obtained during the first 24 hr were discarded and those hatched after 48 hr of incubation were used.

### *Heterodera glycines* J2 hatching from eggs exposed to VOCs emitted by soybean, bean, ryegrass, and alfalfa

*H. glycines* eggs were exposed to VOCs emitted by soybean, bean, ryegrass, and alfalfa. Such species were selected because the literature reported that they produce SCN hatching factors (Fukusawa et al., 1985; Masamune et al., 1987; Riga et al., 2001) (*Glycine max* cv. M6410 IPRO), bean (*Phaseolus vulgaris* cv. Pérola), rye grass (*Lolium multiflorum* cv. BRS Ponteio) and alfalfa (*Medicago sativa* cv. P30) were grown in 121-mL wells of a 72-well Styrofoam tray filled with the artificial substrate (60% pine bark, 15% vermiculite, and 25% humus; Tropstrato®, Mogi Mirim, SP, Brazil) and kept in greenhouse. For testing purposes, vegetable materials (leaves and roots) were collected 30 days after germination, with the exception of ryegrass, which was harvested 60 after germination. Soybean and bean leaves and roots, alfalfa leaves and ryegrass roots were superficially disinfected with 1% sodium hypochlorite for 1 min followed by three washes in distilled water. After drying on paper towels, 1 g of each plant material was placed to one of the polystyrene two-compartment Petri dishes (Fernando et al., 2005) and, in the other compartment was poured 5 ml of an aqueous suspension containing approximately 1200 *H. glycines* eggs. In this assay one individual plant was used for each replicate. The negative control consisted in two-compartment Petri dishes containing only the suspension of eggs in the absence of leaves or roots of the plants. The plates were covered and sealed with polyvinyl chloride (PVC) plastic film and incubated at 25°C in the dark for five days. The evaluation consisted of quantifying the number of J2 hatched five days after the installation of the experiment. For this, the aqueous suspension containing J2 was collected and counted them using a Peters chamber and an inverted light microscope.

### Characterization of the VOCs emitted by soybeans, beans, ryegrass, and alfalfa

Six replicates of treatments were prepared similar to the ones described in the previous item for the characterization of volatile compounds. The samples were added in 20 ml solid phase microextraction (SPME) flasks and kept closed for five days in an incubator at 25°C. The identification of volatile molecules was carried at the Center for Analysis and Chemical Prospecting (Department of Chemistry/UFLA). VOCs were extracted via headspace solid phase microextraction (SPME) (Arthur and Pawliszyn, 1990). The parameters for SPME and gas chromatography-mass spectrometry (GC-MS) were identical to those described in previous work by Gomes et al. (2020).

### *Heterodera glycines* hatching after exposure of eggs to VOCs identified in the gaseous emissions of soybean, bean, ryegrass, and alfalfa

The effect of four VOCs identified on the gaseous emissions of plants leaves and roots was evaluated on the hatching of *H. glycines*. These compounds were chosen according to their availability in the market and costs for acquisition, namely: 3-octanol (99%; Sigma-Aldrich, St. Louis, MO, USA), 1-hexanol (99.5%; Chem Service Inc, West Chester, PA, USA), hexanal (99.3%; Chem Service Inc, West Chester, PA, USA), and linalool (96%; TCI America, Portland, OR, USA). Each of these compounds was used in the J2 hatch test. For this, they were individually diluted in 1% aqueous Tween 80 (0.01 g/ml). Then, a 0.5 ml aliquot of the solution plus 0.5 ml aqueous suspension containing 300 *H. glycines* eggs were placed in 1.5 ml micro tubes. The micro tubes were sealed with PVC plastic film and incubated at 25°C for seven days. Compound solutions were prepared to reach the final concentrations of 200, 600, and 1,000 µg/ml. Water, Tween 80 (1%), and Carbofuran (2,3-dihydro-2,2-dimethyl-1-benzofuran-7-yl N-methyl carbamate; 415 µg/ml) were used as negative control and ZnCl_2_ (3 mM) as positive control. After seven days, the tubes were opened and the number of hatched J2 was quantified. The numbers obtained were used to calculate the hatch percentage for each treatment.

### Nematicidal activity of VOCs against *Heterodera glycines* J2

The VOCs that caused hatching inhibition in the previous trial were selected to assess their toxicity to the J2. For this purpose, was determined the lethal concentration values required to kill 50% of the nematode population (LC_50_) of the compounds 1-hexanol and 3-octanol. The compounds were prepared similarly to that described in the previous item; however, the final concentrations were adjusted to 10, 50, 100, 150, 200, 250, and 300 µg/ml. Then, 0.5 ml of the aqueous solution of each compound plus 0.5 ml of the J2 suspension (300 J2) were placed in 2 ml micro tubes. The micro tubes were sealed with plastic PVC film and incubated at 25°C for 48 hr. After this period, the micro tubes were opened and an aliquot of 100 µl of each replicate was added in holes of 96-well polystyrene plates to determine J2 mortality. The determination of dead nematodes was performed by adding 20 µl of 1 mol/l sodium hydroxide solution (NaOH) to each hole in the plate (Chen and Dickson, 2000). Nematodes that remained immobile after 3 min were considered dead. The J2 mortality percentages obtained in each treatment were used to calculate the LC_50_ of each of the compounds, which represents the concentration capable of killing 50% of the J2 population of *H. glycines*.

### Statistical analysis

The experiments were carried out twice in completely randomized designs with five replicates per treatment in hatching tests, and with six replicates for the lethal concentration experiment. Combined analysis of the experiments (Experiment 1× Experiment 2) was performed and in all tests, it was significant (*p* < 0.05), therefore, the results of each experiment were analyzed separately. Previously, the results of the experiments were subjected to tests of normality (Shapiro–Wilk’s test) and homogeneity of the variance (Bartlett’s test). Thus, the data for each experiment were subjected to the *F* test and when significant (*p* < 0.05) they were subjected to the Scott–Knott’s means test (*p* < 0.05). Mortality percentages were subjected to regression analysis and the determination of the LC_50_ of the compounds was performed. The Sisvar (version 5.6) and SigmaPlot® (12.0) programs were used for statistical and graphical analysis, respectively.

## Results

### *Heterodera glycines* J2 hatching from eggs exposed to VOCs emitted by soybean, bean, ryegrass, and alfalfa

In two experiments, the VOCs emitted by the different plant species induced a significant increase (*p* = 0.0000) in the percentage of *H. glycines* J2 hatched, compared to the negative control. In the first experiment, VOCs released by leaves and roots of soybean and bean provided the largest hatching increases, reaching values up to 71.4% higher than the control. In the same experiment, the volatiles of alfalfa leaves and ryegrass roots also caused significant increases in J2 hatching. In the second experiment, the highest hatching percentage was observed from eggs exposed to the volatiles of soybean roots, being 42.8% higher than the control. The VOCs emitted by other plant species also induced a significant increase in the number of hatched J2 compared to the control (Table 1).

**Table 1.** Percentage of *Heterodera glycines* hatching after exposure of eggs for five days at 25°C to volatile organic compounds emitted by leaves and roots of soybeans and beans; ryegrass roots and alfalfa leaves.

	Hatching (%)			
Treatment	Experiment 1	SD^Z^	Experiment 2	SD^Z^
Control^x^	12.6^y^c	1.58	14.0d	1.23
Alfalfa leaves	18.7b	3.17	17.5b	0.82
Ryegrass roots	18.8b	2.95	15.7c	0.76
Bean roots	19.8a	2.01	18.1b	1.61
Bean leaves	20.6a	1.71	16.4c	0.77
Soybean roots	21.3a	2.49	20.0a	1.24
Soybean leaves	21.6a	1.60	18.6b	2.00

**Notes:** ^x^Control: eggs in water with absence of leaves or roots in the plates; ^y^Means represented by different letters indicate significant differences using the Scott–Knot test (*p* < 0.05); ^Z^SD = standard deviation.

### Characterization of the VOCs emitted by soybean, bean, ryegrass, and alfalfa

GC-MS analysis of the VOCs released by the roots of soybean, ryegrass, and bean revealed the presence of 22, 19, and 17 compounds, respectively. In the emissions of soybean, bean, and alfalfa leaves, 14, 5, and 7 compounds were found, respectively. More compounds were found in soybean and bean roots emissions than in leaves (Table 2). In general, most of the compounds found belong to the classes of alcohols, esters, and ketones, but with incidence of some compounds in the furans, aldehydes, terpenes, pyrazines, and other classes. The compounds 1-hexanol and 2-octanone were detected in all analyzed root emissions (ryegrass, soybean, and bean), but were not identified in the leaf samples. The compounds 2-octanol, 3-methyl-1-butanol, 2-methyl-1-butanol, 6-methyl-5-heptan-2-ol, 2-ethyl-1-hexanol, 2-octanone, 3-octanone, 6-octen-2-one, pentan-3-one, and ethylene benzene were detected only in soybean and bean roots. Some compounds were identified exclusively in certain plant material, such as: heptyl acetate, octyl acetate, isopulegol acetate, n-nonyl acetate, p-ethylanisole, and 4-ethyl-1,2-dimethoxybenzene in ryegrass roots; 3-octanol, lavandulol, 2-butanone, ionone and linalool in soybean leaves; hexanal, b-felandrene, 2-isopropyl-3-methoxypyrazine and 2-sec-butyl-3-methoxypyrazine in soybean roots; and, 1-octen-3-ol, ethyl 2-methylbutyrate, and ethyl butyrate on alfalfa leaves. Three compounds were not identified including two sesquiterpenes. All compounds detected showed low intensity peaks in the samples and, therefore, were categorized only as minor peaks (+).

**Table 2.** Volatile organic compounds from leaves or roots of soybeans, beans, ryegrass, and alfalfa characterized by GC-MS.

Compound	RI exp.^a^	RI lit.^b^	Ryegrass roots	Soybean leaves	Soybean roots	Bean leaves	Bean roots	Alfalfa leaves
*Alcohols*								
Ethanol			+	+	+			+
3-Octanol	1,001	993		+				
2-Octanol	1,005	997			+		+	
3-Methyl-1-butanol	737	734	+	+	+	+	+	
2-Methyl-1-butanol	745	738	+		+	+	+	
1-Hexanol	872	867	+		+		+	
1-Octen-3-ol	984	978						+
6-Methyl-5-hepten-2-ol	1,000	992		+	+		+	
2-Ethyl-1-hexanol	1,032	1,029			+	+	+	
*Esters*								
Ethyl 2-methylbutyrate	845	848						+
Ethyl butyrate	801	802						+
Ethyl acetate	627	623	+					+
Hexyl acetate	1,014	1,014	+				+	
Decyl acetate	1,409	1,408	+					
Heptyl acetate	1,113	1,113	+					
Octyl acetate	1,211	1,211	+					
n-Nonyl acetate	1,310	1,302	+					
*Furans*								
2-Ethylfuran	705	708		+			+	
2-Pentylfuran	990	991	+	+			+	
*Ketones*								
2-Butanone	605	605		+				
2-Pentanone	703	695		+	+			
2-Octanone	991	990	+		+		+	
3-Octanone	988	986		+	+		+	+
6-Octen-2-one	985	985			+		+	
3-Pentanone	704	700		+	+		+	
2-Nonanone	1,092	1,091	+		+			
3,5-Octadien-2-one	1,072	1,068				+	+	
*Aldehydes*								
Hexanal	806	800			+			
*Terpenes*								
Linalool	1,103	1,007		+				
M-Cymene	1,024	1,023	+		+	+		+
B-phellandrene	1,030	1,025			+			
Lavandulol	1,167	1,165		+				
Isopulegol acetate	1,271	1,273	+					
Sesquiterpene*	1,426		+					
Sesquiterpene*	1,432				+			
Ionone	1,482	1,485		+				
*Pyrazines*								
3-Methoxy-2,5-dimethyl	1,051	1,057			+		+	
pyrazine								
2-Isopropyl-3-methoxy	1,089	1,093			+			
pyrazine								
2-Sec-butyl-3-methoxy	1,167	1,164			+			
pyrazine								
*Others*								
P-Ethylanisole	1,114	1,104	+					
Ethenyl-benzene	895	891	+	+	+		+	
4-ethyl-1,2-dimethoxy	1,322		+					
benzene								
Unidentified	1,019				+		+	

**Notes:** ^a^RI exp., Retention rates calculated by the injection of homologous series of alkanes; ^b^RI lit., retention rates according to the literature; +, minor peak; *Not identified. Mass spectrum similar to sesquiterpenes.

### *Heterodera glycines* hatching after exposure of eggs to VOCs identified in the emissions of soybean, bean, ryegrass, and alfalfa

In two experiments, none of the compounds tested at concentrations of 200, 600, and 1000 µg/ml, namely 3-octanol, 1-hexanol, hexanal and linalool, induced J2 hatching at the same level as the positive control (ZnCl_2_). Furthermore, the solubilizing agent used to dissolve the compounds – 1% Tween 80 – when tested alone was more effective in inducing hatching than the solutions containing the compounds. In the second experiment, the solubilizing agent Tween 80 at 1% showed similar hatching percentage (*p* = 0.0000) to the positive control ZnCl_2_.

If, on the one hand, none of the compounds acted as hatching factors, the compounds 3-octanol and 1-hexanol caused, in the three concentrations tested in both assays, a reduction in hatching (*p* < 0.05) similar to that observed with carbofuran (415 µg/ml), which caused the lower percentage of hatching (Table 3).

**Table 3.** Percentage of *Heterodera glycines* J2 hatching from eggs exposed for 7 days to different concentrations of four volatile compounds identified in the emissions of soybean, bean, and ryegrass leaves or roots.

		Hatching (%)	
Treatment	Concentration (µg/mlor mM^x^)	Experiment 1	Experiment 2
Water	–	15.2^y^d	30.2b
Carbofuran	415	3.0f	4.6c
Zinc chloride	3^x^	31.4a	44.4a
Tween 80	–	27.3b	44.6a
Hexanal	1,000	25.3c	31.0b
	600	23.8c	28.0b
	200	22.7c	30.1b
Linalool	1,000	13.3d	26.0b
	600	15.3d	25.0b
	200	16.8d	28.4b
1-Hexanol	1,000	2.9f	5.3c
	600	1.9f	4.1c
	200	3.4f	5.5c
3-Octanol	1,000	3.8f	5.0c
	600	3.3f	8.3c
	200	7.5e	8.8c

**Note:** ^y^Means represented by different letters indicate significant differences using the Scott–Knot test (*p* < 0.05).

### Nematicidal activity of VOCs against *Heterodera glycines* J2

**Figure 1:**
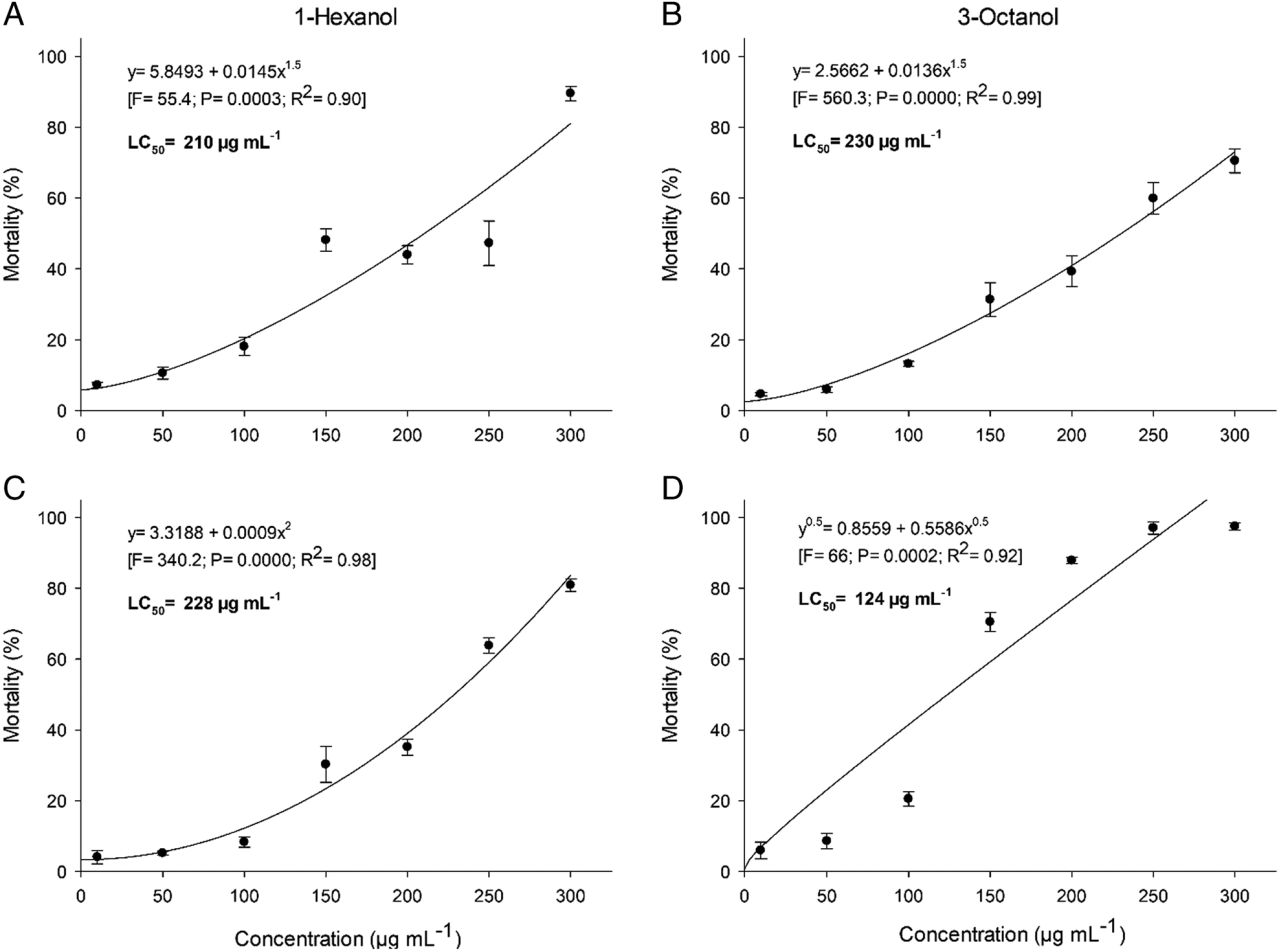
Mortality and lethal concentration (LC_50_) of second-stage juveniles of *Heterodera glycines* exposed to different concentrations of 1-hexanol and 3-octanol in experiments 1 (A and B) and 2 (C and D). Bars indicate the standard error of the mean.

In two assays, the increase of 1-hexanol and 3-octanol concentrations caused increase in *H. glycines* J2 mortality. Through regression analysis, the LC_50_ values of compounds 1-hexanol and 3-octanol were 210 and 228 µg/ml, respectively, in the first experiment and, 230 and 124 µg/ml in the second one (Fig. 1).

## Discussion

The hatching induction of *H. glycines* J2 was observed from extracts and exudates from soybean, ryegrass, alfalfa, and bean roots (Riga et al., 2001). With the present work, we highlighted the effect of short-chain molecules released by these plants as SCN hatching factors. Here, we test the effect of VOCs only on dispersed eggs. However, approximately 2/3 of *H. glycines* eggs remain inside the cysts until the right time for hatching. As VOCs easily cross biological membranes, we do not believe that cysts are able to inhibit the effect of VOCs on the eggs within them. In addition, cysts have openings that are also entrance for VOCs. Although no molecules have been characterized as hatching factor. The diversity of volatile molecules identified by GC-MS creates a catalog of molecules that can be investigated in the future to find an effective SCN hatching factor.

In this work, the identification of VOCs released by plants though GC-MS allowed the investigation of the interaction of some VOCs with the SCN. Four molecules, 3-octanol, 1-hexanol, hexanal and linalool were selected for more in-depth tests of hatching stimulus. While, none of them acted as hatching inducer, two of them 3-octanol and 1-hexanol caused hatching inhibition. We should emphasizing that this result occurred from the three concentrations test. Therefore, the possible action of these molecules as SCN hatching inducer when in different concentrations cannot be excluded. In addition, the synergistic effect between the compounds was also not evaluated. Indeed, our results strongly suggest that a synergistic mechanism is in place and that a more complex mixture of the compounds is needed to trigger the SCN hatching than just one single compound. The synergistic effect of different compounds on the hatching of cyst nematodes has been reported in the literature. Potato root leachate contain multiple hatching factors that exhibit a synergistic effect with one another, as seen in purified isolates stimulating lower hatch compared to the crude (Devine and Jones, 2000b). Thus, from the catalog of molecules presented here, further research can be carried out seeking to identify an individual molecule or a mixture of compounds that have the characteristic of SCN hatching factor.

In recent years, studies have revealed VOCs emitted by plant residues with nematicidal activity similar to commercial nematicides (Gomes et al., 2020; Silva et al., 2018). In the present study, several compounds identified in plant emissions, such as 3-methyl-1-butanol, 2-methyl-1-butanol, 3-pentanone, 2-nonanone, linalool, m-cymene, 2-octanol, 2-ethyl-1-hexanol, ethyl acetate, 2-ethylfuran and 2-octanone have proven nematicidal activity (Aissani et al., 2015; Gu et al., 2007; Hewavitharana et al., 2014; Sharma et al., 2018; Zhai et al., 2018). In this sense, the compounds 3-octanol detected in soybean leaves and 1-hexanol detected in soybean, bean, and ryegrass roots, were toxic to eggs and *H. glycines* J2. The nematicidal activity of these compounds had already been reported for other plant-parasitic nematodes (Silva, 2015). The toxicity of 3-octanol has also been reported for *Drosophila melanogaster*, being lethal at a concentration of 14.7 µl/ml (Inamdar et al., 2010). The essential oil of *Origanum vulgare* (Lamiaceae), which has nematicidal activity in *Bursaphelenchus xylophilus*, showed the presence of 3-octanol (1%), however, the isolated effect of this alcohol was not investigated by the authors (Barbosa et al., 2010).

Interestingly, Tween 80 (negative control) used for dissolving VOCs stimulated hatching at a value similar to ZnCl_2_ (positive control). The change in eggshell permeability may be a central point in the hatching of cyst nematodes (Perry and Trett, 1986). In studies with lepidopterans such as *Galleria mellonella*, Tween 80 is used as a permeabilizer in the egg cryopreservation process (Abidalla and Roversi, 2018; Cosi et al., 2010; Roversi et al., 2008). Thus, the effect of Tween 80, observed in the present study, may also be related to the permeability of the eggshell of *H. glycines*, however, further research is necessary to clarify this information.

Having a more comprehensive knowledge of the chemical constituents of plant diffusates will lead to our better understanding of the interactions between *H. glycines* and their host plants, thus contributing to the development of strategies to control this important plant-parasitic nematode. Here, we reveal that short-chain molecules released by different plant species act as *H. glycines* hatching factors. At the same time, some of these compounds, depending on their concentration, may have nematicidal activity.

**Supplementary Table 1.** Raw data related to Table 1.

Treatments	Time	Total_J2_hatched	%_J2_hatched
Control	1	147	13.01
Control	1	125	11.06
Control	1	169	14.96
Control	1	127	11.24
Control	1	145	12.83
Control	2	159	13.25
Control	2	183	15.25
Control	2	177	14.75
Control	2	147	12.25
Control	2	175	14.58
Soybean roots	1	277	24.51
Soybean roots	1	224	19.82
Soybean roots	1	203	17.96
Soybean roots	1	250	22.12
Soybean roots	1	249	22.04
Soybean roots	2	218	18.17
Soybean roots	2	258	21.50
Soybean roots	2	235	19.58
Soybean roots	2	244	20.33
Soybean roots	2	245	20.42
Bean roots	1	218	19.29
Bean roots	1	219	19.38
Bean roots	1	227	20.09
Bean roots	1	258	22.83
Bean roots	1	195	17.26
Bean roots	2	185	15.42
Bean roots	2	234	19.50
Bean roots	2	220	18.33
Bean roots	2	230	19.17
Bean roots	2	219	18.25
Ryegrass roots	1	209	18.50
Ryegrass roots	1	169	14.96
Ryegrass roots	1	234	20.71
Ryegrass roots	1	254	22.48
Ryegrass roots	1	194	17.17
Ryegrass roots	2	200	16.67
Ryegrass roots	2	189	15.75
Ryegrass roots	2	186	15.50
Ryegrass roots	2	175	14.58
Ryegrass roots	2	192	16.00
Soybean leaves	1	236	20.88
Soybean leaves	1	224	19.82
Soybean leaves	1	273	24.16
Soybean leaves	1	243	21.50
Soybean leaves	1	242	21.42
Soybean leaves	2	261	21.75
Soybean leaves	2	195	16.25
Soybean leaves	2	223	18.58
Soybean leaves	2	215	17.92
Soybean leaves	2	220	18.33
Bean leaves	1	215	19.03
Bean leaves	1	217	19.20
Bean leaves	1	250	22.12
Bean leaves	1	256	22.65
Bean leaves	1	223	19.73
Bean leaves	2	198	16.50
Bean leaves	2	197	16.42
Bean leaves	2	192	16.00
Bean leaves	2	186	15.50
Bean leaves	2	211	17.58
Alfalfa leaves	1	184	16.28
Alfalfa leaves	1	172	15.22
Alfalfa leaves	1	203	17.96
Alfalfa leaves	1	251	22.21
Alfalfa leaves	1	246	21.77
Alfalfa leaves	2	217	18.08
Alfalfa leaves	2	209	17.42
Alfalfa leaves	2	216	18.00
Alfalfa leaves	2	214	17.83
Alfalfa leaves	2	193	16.08

**Notes:** 1 exp with 1,130 eggs; 2 exp with 1,200 eggs.
